# Self‐compassion improves barrier self‐efficacy and subsequently physical activity: A test of longitudinal mediation using a representative sample of the United Kingdom

**DOI:** 10.1111/bjhp.12757

**Published:** 2024-10-18

**Authors:** Shuge Zhang, Andy Pringle, Clare Roscoe

**Affiliations:** ^1^ Hunan University of Technology Zhuzhou China; ^2^ University of Derby Derby UK

**Keywords:** compassion, longitudinal mediation, panel data, physical activity, self‐efficacy

## Abstract

**Introduction:**

Self‐compassion, which directs the awareness of suffering, sympathetic concerns and caring motives towards oneself, is an important psychological quality and resource for health and well‐being. In the context of physical activity, self‐compassion can help individuals overcome obstacles, recuperate from a setback or a lapse and engage in regular physical activity. The present research was the first to examine the longitudinal effects of self‐compassion on physical activity and the mediation role of barrier self‐efficacy of such effects.

**Methods:**

We recruited a national representative sample of 654 UK adults and followed them over three timepoints across 9 months. At each time point, participants completed an online survey assessing levels of state self‐compassion, barrier self‐efficacy and physical activity behaviours. We examined the longitudinal effects of self‐compassion on physical activity and the mediation role of barrier self‐efficacy.

**Results:**

Baseline state self‐compassion consistently correlated with physical activity levels at Times 2 and 3. Barrier self‐efficacy at Time 2 mediated the longitudinal effect of baseline state self‐compassion on Time 3 physical activity, after controlling for within‐ (e.g., Time 1 on Time 2 self‐compassion) and between‐person variations (e.g., covariance of self‐compassion and physical activity within a timepoint).

**Conclusions:**

Adopting a self‐compassionate mind facilitates engagement and maintenance of physical activity. Future studies could consider accelerometer‐based physical activity measures and develop and validate a more context‐specific state self‐compassion measure tailored for physical activity contexts. Researchers and practitioners should consider incorporating self‐compassion to future interventions and education programmes for promoting physical activity.

Compassion is often understood via its connection to the mammalian attachment system in the form of a stimulus–response algorithm (e.g., parents' recognition of a child's emotion and response to it with remedial and soothing behaviours) and thus is commonly known as a sensitivity to sufferings with a commitment to try to alleviate it (Gilbert, 2015, 2020). A specific form of compassion that directs the awareness of sufferings, sympathetic concerns and caring motives towards oneself, namely self‐compassion, has been found to play a critical role in one's health and wellbeing (see Neff, 2023c, for a review). In its original conceptualization (Neff, 2003a), self‐compassion encapsulates three central components, including *self‐kindness* (e.g., being tolerant of one's flaws and inadequacies), *common humanity* (e.g., seeing failures or other negative experiences as part of the shared human condition) and *mindfulness* (e.g., taking a balanced view and keeping situations in perspective). Such a compassionate mind and its related attributes can be cultivated and enhanced via proper training and practices (Gilbert & Van Gordon, 2023; Mosewich, 2020; Walton et al., 2022) and have a wide range of benefits, such as more adaptive self‐regulation (Terry & Leary, 2011), enhanced coping under difficulties (Barczak & Eklund, 2020), greater prosociality (Marshall et al., 2020) and psychological wellbeing (Maner et al., 2023).

However, knowledge is relatively scarce as research to date has yet to receive ample attention regarding the extent to which and how self‐compassion influences one's health behaviours such as physical activity. Within the limited existing studies examining self‐compassion in the context of physical activity, there are also many limitations (e.g., cross‐sectional design, insufficient understanding of mechanism) that constrain the implications of this work (see Hall et al., 2023). As such, the current research was set to examine the longitudinal influence of self‐compassion on physical activity behaviours and to test the potential mechanism of barrier self‐efficacy underlying such an effect, based on prior knowledge of the relationship between self‐compassion and self‐efficacy (see Liao et al., 2021, for review) and between self‐efficacy and physical activity (see Biddle et al., 2011, for review).

## Self‐compassion in the context of physical activity

Literature has generally supported the relationship between self‐compassion and physical activity behaviour. A recent systematic review by Hall et al. (2023), involving 10 studies of 6808 participants, suggested a positive and small‐to‐moderate correlation between physical activity and self‐compassion. Nevertheless, Wong et al.'s (2021) meta‐analytical work of intervention studies argued that the correlation of physical activity and self‐compassion is typically driven by the causality of physical activity; that is, physical activity interventions consistently generated improved self‐compassion outcomes. While Hall et al.'s (2023) and Wong et al.'s (2021) work further enriched the evidence of psychological benefits associated with physical activity, it doesn't provide a sound framework to explain the causality between physical activity and self‐compassion and has overlooked the potential role of self‐compassion as an antecedent or driver for one's engagement and maintenance in physical activity.

Given the well‐documented barriers and challenges (e.g., lack of motivation and support, negative emotions, time capacity issues, accessibility) that individuals can encounter when attempting to engage in or maintain physical activity (see Biddle et al., 2011; Bodde & Seo, 2009; Rhodes et al., 2009; Sit et al., 2008; for review), Zhang et al. (2023) suggested that self‐compassion is a useful approach to physical activity because it provides emotional benefits (e.g., less distressful, more accepting minds towards exercise relapse and other common difficulties in relation to physical activity) and adaptive coping or regulation resources as compensation to facilitate the engagement and maintenance of physical activity (e.g., allowing oneself to slow down, recuperate and return to physical activity after an exercise‐related setback such as dissatisfaction or demotivation due to not achieving desired outcomes). In their study of a British adult sample, Zhang et al. (2023) found a small but positive cross‐sectional effect of self‐compassion on moderate‐to‐vigorous physical activity levels, but such an effect was not evident once controlled for physical activity covariates such as psychological distress and barrier self‐efficacy. Similar findings were reported from Canadian (Semenchuk et al., 2021) and Australian (Hallion et al., 2019) samples, that individuals high in self‐compassion appeared to achieve greater levels of physical activity, but such a tendency was not significant after controlling for relevant psycho‐behavioural factors such as emotional reactions and health‐promoting behaviours (Semenchuk et al., 2021) and other individual differences such as social demographics and self‐efficacy (Hallion et al., 2019).

Besides the partial empirical support of self‐compassion's role in promoting physical activity behaviours demonstrated in Hallion et al. (2019), Semenchuk et al. (2021) and Zhang et al.'s (2023) work, we see several limitations in these preliminary studies that require addressing. For instance, all these works adopted *Self‐Compassion Scale*‐based measures (i.e., Neff, 2003b; Raes et al., 2011) which are designed to capture the dispositional and trait‐like aspects of self‐compassion. However, self‐compassion has a state‐like component (Neff et al., 2021) and can be applied as a skill or strategy in certain circumstances to facilitate self‐regulation and coping (Gilbert & Van Gordon, 2023; Neff, 2023c). The state conceptualization of self‐compassion (i.e., Neff et al., 2021) is particularly relevant to the adoption and maintenance of physical activity which is considered a dynamic context full of varying drivers, stressors and demotivators (Nigg et al., 2008). This is because drivers (e.g., self‐efficacy and motivation) and barriers (e.g., stress and setbacks) of one's physical activity behaviours can fluctuate over time in an individual (Nigg et al., 2008) and state self‐compassion can be triggered under suffering occurring instances or difficult situations (Neff et al., 2021) thus facilitate an individual to establish or maintain self‐efficacy and overcome barriers to physical activity (Zhang et al., 2023). As such, research on self‐compassion and physical activity should focus more on the relatively overlooked state aspects of self‐compassion.

Moreover, a lack of longitudinal, causal design limited the value and implications of existing works on self‐compassion and physical activity (e.g., failed to inform if self‐compassion manifests a causal effect on physical activity). As such, in the present research, we aimed to examine the longitudinal effect of one's state self‐compassion towards physical activity on their physical activity levels.

## Barrier self‐efficacy: A potential mediating mechanism

Despite the limitations, literature has suggested that the self‐regulation benefit, especially in the form of enhanced self‐efficacy (i.e., one's efficacious belief in executing a certain action that is context‐specific, task‐related and can be independent of one's actual ability and self‐appraisal; see Bandura, 1977, 1997), accounts for why self‐compassion is facilitative to the adoption and maintenance of physical activity behaviours. Research has found that individuals high in self‐compassion reported greater levels of intrinsic motivation and lower levels of external regulation (e.g., using rewards to drive oneself for exercising) (Semenchuk et al., 2018). These individuals also demonstrate superiority in maintaining motivation when experiencing exercise lapses or setbacks (Signore et al., 2021). Moreover, self‐compassion is associated with lower levels of self‐stigmatizing of one's weight and body image (Cox et al., 2019; Huellemann et al., 2023). From a social cognitive perspective (see Bandura, 1997, 2004), these motivational and emotional regulation benefits associated with self‐compassion should strengthen one's efficacious belief and thus greater physical activity. Systematic reviews also support a positive relationship between self‐compassion and self‐efficacy in both general (Liao et al., 2021) and physical activity (Biber & Ellis, 2019) settings.

Built on these prior findings and knowledge, Zhang et al. (2023) proposed that a domain‐specific self‐efficacy, namely barrier self‐efficacy (i.e., one's efficacious belief in overcoming barriers for engaging in physical activity; see Lewis et al., 2016; Marcus et al., 1992; Zhang et al., 2021), is a promising candidate that can explain self‐compassion's influences on physical activity (or mediate the self‐compassion‐physical activity relationship). These researchers found that self‐compassion manifested a significant and positive indirect effect on moderate‐to‐vigorous physical activity via increased barrier self‐efficacy. This observed indirect effect consisted of a small‐to‐moderate and positive effect of self‐compassion on barrier self‐efficacy and a moderate‐to‐large effect of barrier self‐efficacy on physical activity. The findings typically provided support to the proposition that self‐compassion is useful in facilitating oneself to cope with difficulties and overcome barriers for engaging and maintaining desired physical activity. However, as discussed earlier, Zhang et al.'s (2023) work is limited by its cross‐sectional design and the trait‐like measure of self‐compassion.

## The present research

Given the potential of self‐compassion in promoting physical activity and the identified gaps (e.g., lack of longitudinal or causal evidence, trait‐not state‐like measure of self‐compassion), we aimed to examine the longitudinal effect of state self‐compassion (especially that in relating to physical activity) on one's physical activity behaviours. More importantly, we set the current study to test the mediating role of barrier self‐efficacy underlying the effect of self‐compassion on physical activity. Since a robust mediating effect is best examined using a longitudinal or interventional design whilst controlling for potential confounders (see Rohrer et al., 2022), we adopted a three‐wave panel data design to test the mediation of barrier self‐efficacy within the effect of self‐compassion on physical activity. Such a longitudinal panel data design (i.e., all study variables measured repeatedly and included for analysis at all data collection times) can offer useful insights into causal interference (see Finkel, 2020), because panel data simultaneously control for within‐person (e.g., changes of self‐compassion, barrier self‐efficacy and physical activity over time) and between‐person (e.g., covariance of self‐compassion, barrier self‐efficacy and physical activity at each time point) variations when conducting longitudinal analysis. In other words, a lagged effect (e.g., the effect of a variable on another variable at a delayed time or a later time point) in a panel data model can provide causal interference as such an effect has controlled for both within‐person changes and between‐person differences so that any change in the second variable at a delayed time would be accounted by variations in the other variable at an earlier time point (see also Zhang, 2014). We hypothesized that state self‐compassion at baseline or Time 1 contributes to increased barrier self‐efficacy at Time 2 which subsequently enhances physical activity at Time 3 after controlling for within‐ and between‐person variations; that is, a positive, significant indirect effect of self‐compassion on physical activity via enhanced barrier self‐efficacy (i.e., the mediating mechanism).

## METHODS

### Participants

We recruited a representative sample involving 654 UK adults (Mean_age_ = 40.84 years, SD = 13.59; 49.5% females) from Prolific (i.e., the UK's largest research participants crowd‐sourcing platform; https://www.prolific.com/). The location of participants covered all areas of the UK, with the top three areas being *South East England* (15.7%), *North West England* (11.3%) and *South West England* (10.1%), whilst the bottom three areas being *Northern Ireland* (2.1%), *Wales* (5.2%) and *North East England* (5.4%). Among these participants, 86.7% were *White British*, 6.5% were *Asian or Asian British*, 3.2% were *Black, Caribbean, African or Black British*, 2.4% were *Mixed*, with the rest being other ethnic groups. 73% of the participants^1^ completed all three waves of data collection over a nine‐month period (i.e., each data collection window lasted for 1 month, with a 3‐month interval in between two data collection timepoints). A priori power analysis for longitudinal mediation using continuous three‐wave panel data via Monte Carlo simulation (see Zhang, 2014) suggested that the sample is sufficient to detect a relatively small longitudinal mediation (standardized indirect effect = .02, power = .92) or a lagged effect (standardized coefficient = .15, power = .97) at .05 alpha.^2^

### Measures

#### State self‐compassion

We adapted the *State Self‐Compassion Scale* (SSCS; Neff et al., 2021) to assess participants' self‐compassionate mind^3^ in relation to physical activity. Specifically, we instructed participants to think about any physical activity‐related situation(s) they were experiencing at the time of data collection that might be painful, difficult, challenging, or made them feel inadequate in some way,^4^ before rating their feelings towards each SSCS statement (e.g., “I'm giving myself the caring and tenderness I need”, “I'm taking a balanced view of this painful situation”). All SSCS items (18 in total) were rated on a 5‐point Likert scale from 1 (*not at all true for me*) to 5 (*very true for me*). Following Neff et al.'s suggestion, we took a mean score for all the SSCS items after proper reverse‐coding for further analysis so that higher scores indicate greater self‐compassion in relation to physical activity.

### Barrier self‐efficacy

We employed Marcus et al.'s (1992) *Self‐Efficacy Inventory* (SEI) to examine participants' efficacious belief that they can confidently engage in physical activity despite the varied barriers they could experience (e.g., “When I am tired”, “When I am in a bad mood”). We asked participants to report their feelings at the time of data collection regarding each challenging condition stated in the SEI items (5 in total). All SEI items were rated on an 11‐point Likert scale from 0 (*0% confident, not confident at all*) to 10 (*100% confident, very much confident*). We aggregated participants' responses for all items to mean scores for further analysis, with a larger score reflecting greater confidence in overcoming barriers or challenging situations to physical activity engagement.

### Physical activity

We adopted the *International Physical Activity Questionnaire – Short Form* (IPAQ‐SF; Maddison et al., 2007) to track participants' levels of physical activity and sedentary behaviour during the study period. Following guidance (van Poppel et al., 2010), we used the IPAQ‐SF to assess averaged *duration* (i.e., hours and minutes per day) and *frequency* (i.e., days per week) of vigorous, moderate and light physical activity as well as sedentary behaviour providing standard instructions and examples (see Maddison et al., 2007). We calculated participants' daily average time (min/day) spent in moderate‐to‐vigorous physical activity (MVPA), light physical activity (LPA) and sedentary behaviours (SBs) for further analysis. Such an approach (i.e., analysing MVPA, LPA and SB separately) is desirable as it can capture the dynamic changes of the different physical activity components at a within‐person level over the 9 months study time.

### Procedures

With ethics approval, we created an online survey for baseline (Time 1) using Qualtrics and advertised it via Prolific. We used the built‐in function of representative sample^5^ in the Prolific to screen and recruit trustworthy participants (i.e., active within 90 days prior to the start of data collection, no history of blacklist or complaint by other researchers) who were UK citizens and healthy (i.e., not receiving any medication/treatment or in the condition that might prevent one from engaging in physical activity) at the time of baseline data collection. We provided all study information (e.g., the longitudinal nature of the study) at the time of recruitment and requested the completion of an online consent before one could formally partaking in the study via an online survey link. We thanked and debriefed each participant at the end of the online survey and offered £1 incentive via Prolific as compensation.^6^ We repeated these processes in the 5th (Time 2) and the 9th (Time 3) month since the launch of the three‐wave data collection.

### Data analysis

**FIGURE 1:**
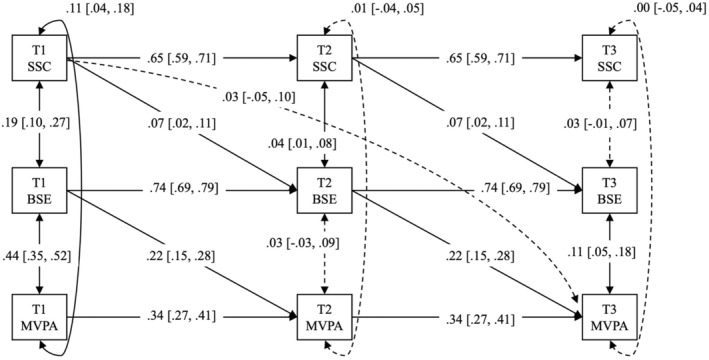
Panel analysis of longitudinal mediation of barrier self‐efficacy (BSE) in the effect of state self‐compassion (SSC) on daily moderate‐to‐vigorous physical activity (MVPA) over three timepoints across 9 months (T1, T2, T3). Dotted line indicates a non‐significant effect, whilst the solid line indicates a significant effect. All effects are standardized, with a 95% confidence interval presented in the bracket.

**FIGURE 2:**
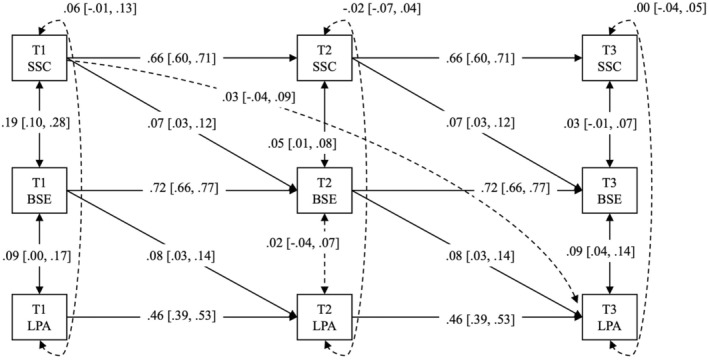
Panel analysis of longitudinal mediation of barrier self‐efficacy (BSE) in the effect of state self‐compassion (SSC) on daily light physical activity (LPA) over three timepoints across 9 months (T1, T2, T3). Dotted line indicates a non‐significant effect, whilst the solid line indicates a significant effect. All effects are standardized, with a 95% confidence interval presented in the bracket.

**FIGURE 3:**
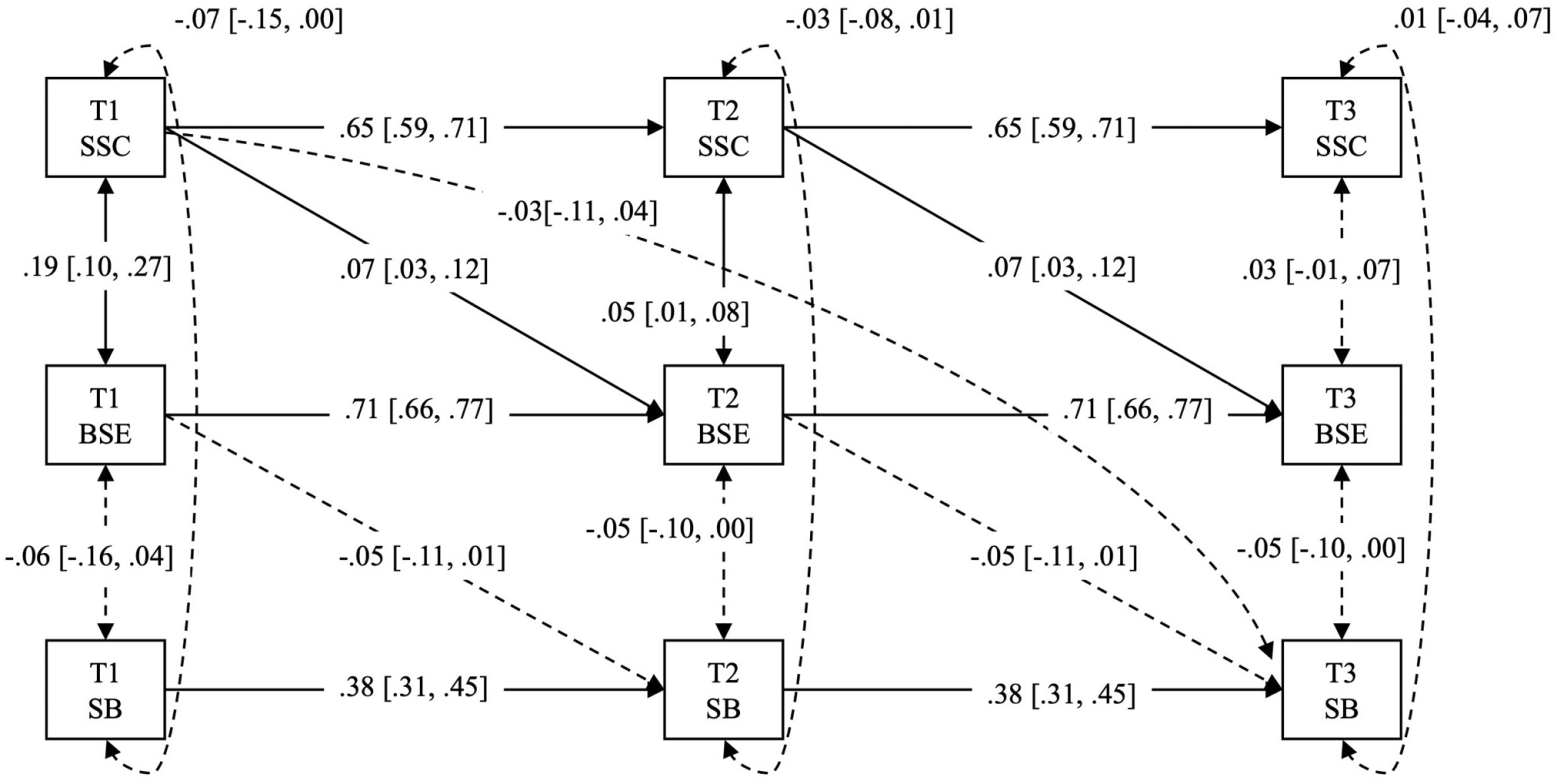
Panel analysis of longitudinal mediation of barrier self‐efficacy (BSE) in the effect of state self‐compassion (SSC) on daily sedentary behaviour (SB) over three timepoints across 9 months (T1, T2, T3). Dotted line indicates a non‐significant effect, whilst the solid line indicates a significant effect. All effects are standardized, with a 95% confidence interval presented in the bracket.

For the preliminary analysis, we checked missing data, generated descriptive statistics and assessed zero‐order correlations as well as the Cronbach's alpha for all study variables in SPSS Version 28. For the main analysis, we tested three longitudinal mediation models on MVPA, LPA and SB, respectively, using Mplus Version 8 (Muthén & Muthén, 2017). Specifically, we modelled Time 1 self‐compassion as the exposure or predictor variable, Time 2 barrier self‐efficacy as the mediator and Time 3 MVPA/LPA/SB as the outcome variable, thus enabling the test of the Time 1 self‐compassion's indirect effect on Time 3 physical activity outcomes. Importantly, we specified autoregressive paths for all study variables (e.g., the effect of self‐compassion at Time 1 on Time 2 and at Time 2 on Time 3) to account for within‐person changes (i.e., variability) and trait‐like components (i.e., stability) of each study variable across the three study timepoints. We also controlled for synchronous correlations of all study variables at each time point when testing the hypothesized longitudinal mediations. We further built the lagged effect of Time 1 barrier self‐efficacy on Time 2 physical activity outcomes and Time 2 self‐compassion on Time 3 barrier self‐efficacy for more precise estimation of Time 1 self‐compassion's effect on Time 3 physical activity outcomes via Time 2 barrier self‐efficacy (see Zhang, 2014 for more guidance on testing longitudinal mediation). Figures 1, 2, 3 illustrated the three longitudinal mediation models we conceptualized and tested.

When testing the hypothesized longitudinal mediation, we used the robust maximum likelihood estimator (i.e., MLR in Mplus) for more accurate parameter estimation and to mitigate the potential influence of data non‐normality. The MLR estimator in Mplus also enables the Full Information Maximum Likelihood approach (FIML; Hirose et al., 2015). According to Hirose et al. (2015), the FIML method does not require missing completely at random and is reliable in addressing up to 50% or more missing in endogenous variables (e.g., Time 2 and 3 variables in our study) given no missing in any exogenous variables (e.g., Time 1 variables in our study). The use of the FIML method, therefore, was desirable for this study (i.e., only 27% missing in Time 2 and 3), as it could retain the participants with missing data or dropped out at Time 2 or 3 for model estimation, thus maintaining optimal statistical power and preventing loss of information. In other words, despite there were only 475 participants at Time 2 and 3 due to dropouts from the 654 Time 1 baseline participants, the FIML method enabled us to retain all 654 participants from the baseline for the longitudinal mediation analysis whereas the missing values at Times 2 and 3 were estimated based on distribution assumptions. Such a method has been validated and recommended for dealing with missing data (see also Newman, 2014).

To understand the extent to which the hypothesized longitudinal mediation models can explain the observed data, we adopted Hu and Bentler's (1999) recommendations for assessing model fit. In particular, we used Chi‐square^7^ (*χ* ^2^), comparative fit index (CFI), standardized root mean square residual (SRMR) and the root mean square error of approximation (RMSEA) to assess good model fit, with close to .95 or larger for CFI and close to .08/.06 or smaller for SRMR/RMSEA indicating good fit.

For the longitudinal mediation analysis, we report standardized coefficient (*β*), 95% confidence intervals (CIs) and precise *p*‐value to two decimal points for each of the autoregressive (e.g., self‐compassion at Time 1 on Time 2) and lagged/direct (e.g., Time 1 self‐compassion on Time 2 barrier self‐efficacy). Such an approach allowed us to generate a completely standardized estimate to provide a common metric for assessing the magnitude of the indirect effect of Time 1 self‐compassion on Time 3 physical activity outcomes (i.e., the mediation). Following Cohen (1988), we considered .10, .30 and .50 standardized regression coefficients as small, medium and large, respectively, for a lagged/direct effect, which informs .01, .09 and .25 as small, medium and large, respectively, for an indirect effect.^8^

## RESULTS

### Preliminary analysis

No missing data were found at baseline, whilst 27% missing were found in all Time 2 and 3 variables (due to dropouts from Time 1 to Time 2; no dropouts from Time 2 to 3). For all study variables from all timepoints, maximum skewness was 1.30 (Time 1 LPA) and maximum kurtosis was 1.55 (Time 1 MVPA), which fulfils the requirements for running path models (i.e., within ±3 for skewness and within ±10 for kurtosis; see Kline, 2016). Cronbach's alphas achieved .81–.84 for self‐compassion and .88–.89 for barrier self‐efficacy measures, indicating very good internal reliability. For synchronous correlations (i.e., the correlations of study variables within each timepoint), self‐compassion consistently manifested small‐to‐medium correlations with barrier self‐efficacy (*r* = .20–.24) but not with physical activity outcomes except for Time 1 MVPA (*r* = .12). Barrier self‐efficacy consistently manifested medium‐to‐large correlations with MVPA (*r* = .38–.45) and small‐to‐medium correlations with LPA (*r* = .12–.24). The correlation between self‐compassion and barrier self‐efficacy was small‐to‐medium at all timepoints (*r* = .20–.24). More importantly, for predictive correlations (i.e., correlations of one variable at an earlier timepoint and another variable at a later timepoint), self‐compassion at Time 1 correlated consistently to MVPA and LPA at Times 2 and 3 (*r* = .10–.11). Meanwhile, barrier self‐efficacy at Time 1 correlated consistently to MVPA (*r* = .36–.42) and LPA at Times 2 and 3 (*r* = .14–.17). Table 1 displays all details of the descriptive statistics and zero‐order correlations between study variables.

**TABLE 1:** Descriptive statistics and zero‐order correlations of study variables.

	Mean	SD	T1	T1	T1	T1	T1	T2	T2	T2	T2	T2	T3	T3	T3	T3	T3
			SSC	BSE	MVPA	LPA	SB	SSC	BSE	MVPA	LPA	SB	SSC	BSE	MVPA	LPA	SB
T1 SSC	3.30	.83	(.81)														
T1 BSE	5.61	2.29	.20**	(.89)													
T1 MVPA	26.22	26.08	.12**	.45**	–												
T1 LPA	36.61	29.75	.08	.10*	.28**	–											
T1 SB	427.72	204.02	−.07	−.07	−.07	−.15**	–										
T2 SSC	3.37	.86	.76**	.18**	.10*	.03	−.06	(.82)									
T2 BSE	4.14	1.51	.23**	.76**	.44**	.14**	−.10*	.24**	(.88)								
T2 MVPA	30.17	28.30	.10*	.42**	.46**	.23**	−.12**	.09	.38**	–							
T2 LPA	36.68	29.53	.11*	.14*	.22**	.54**	−.13**	.06	.15**	.28**	–						
T2 SB	419.57	205.38	−.04	−.09	−.05	−.14**	.46**	−.07	−.13**	−.09	−.15**	–					
T3 SSC	3.23	.86	.72**	.14**	.11*	.07	−.04	.72**	.18**	.07	.06	−.03	(.84)				
T3 BSE	4.14	1.56	.23**	.58**	.44**	.16**	−.19**	.23**	.71**	.38**	.15**	−.16**	.20**	(.89)			
T3 MVPA	27.39	26.39	.11*	.36**	.44**	.17**	−.12**	.10*	.33**	.49**	.20**	−.07	.08	.40**	–		
T3 LPA	37.56	29.99	.10*	.17**	.24*	.55**	−.12**	.05	.17**	.25**	.57**	−.11*	.07	.24**	.30**	–	
T3 SB	425.60	204.34	−.06	−.11*	−.06	−.15**	.45**	−.07	−.11*	−.11*	−.10*	.45**	−.04	−.19**	−.12**	−.17**	–

*Note*: The range of score is 1–5 for SSC (state self‐compassion) and 0–10 for BSE (barrier self‐efficacy). The unit is *minutes per day* for MVPA (moderate‐to‐vigorous physical activity), LPA (light physical activity), and SB (sedentary behaviour). T1, T2, and T3 refer to Time 1, 2 and 3, respectively, with a 3‐month time interval in each pair of contiguous timepoints. Cronbach's alpha of a study measure (when appropriate) is displayed within the parenthesis.

* *p* < .05.

** *p* < .01.

### Main analysis

For moderate‐to‐vigorous physical activity (MVPA), the longitudinal mediation model (see Figure 1) explained 24.5% variance in its changes over the study period (*χ* ^2^ = 90.13, *df* = 18, *p* = .00; CFI = .96, RMSEA = .07, SRMR = .05). After adjusting for autoregressive effects and synchronous correlations of all study variables,^9^ Time 1 self‐compassion manifested a small but positive effect on Time 2 barrier self‐efficacy (*β* = .07, *p* = .00; 95% CI [.02, .11]), of which the latter (i.e., Time 2 barrier self‐efficacy) exerted a small‐to‐medium and positive effect on Time 3 MVPA (*β* = .22, *p* = .00; 95% CI [.15, .28]). Despite a lack of direct effect of Time 1 self‐compassion on Time 3 MVPA (*β* = .03, *p* = .56; 95% CI [−.05, .10]), importantly, the indirect effect of Time 1 self‐compassion on Time 3 MVPA via Time 2 barrier self‐efficacy was positive and significant (standardized estimate = .02, *p* = .01; 95% CI [.01, .03]), indicating a statistically meaningful mediation of barrier self‐efficacy.

For comparison, the longitudinal mediation model (see Figure 2) explained 32.8% variance in the changes in light physical activity (LPA) over the study period (*χ* ^2^ = 67.01, *df* = 18, *p* = .00; CFI = .97, RMSEA = .06, SRMR = .05). To expand, after adjusting for autoregressive effects and synchronous correlations of all study variables, Time 1 self‐compassion manifested a small but positive effect on Time 2 barrier self‐efficacy (*β* = .07, *p* = .00; 95% CI [.02, .11]), of which the latter (i.e., Time 2 barrier self‐efficacy) also exerted a small but positive effect on Time 3 LPA (*β* = .08, *p* = .00; 95% CI [.03, .14]). More importantly, the indirect effect of Time 1 self‐compassion on Time 3 LPA via Time 2 barrier self‐efficacy was positive and significant (standardized estimate = .01, *p* = .03; 95% CI [.00, .02]), suggesting a statistically meaningful mediation. Nevertheless, Time 1 self‐compassion did not manifest a significant direct effect on Time 3 LPA (*β* = .03, *p* = .42; 95% CI [−.04, .09]).

Regarding time spent in sedentary behaviour, the longitudinal mediation model (see Figure 3) explained 21.7% variance in its changes over the study period (*χ* ^2^ = 70.97, *df* = 18, *p* = .00; CFI = .97, RMSEA = .06, SRMR = .04). After adjusting for autoregressive effects and synchronous correlations of all study variables, Time 1 self‐compassion manifested a small but positive effect on Time 2 barrier self‐efficacy (*β* = .07, *p* = .00; 95% CI [.02, .11]), but Time 2 barrier self‐efficacy did not impact Time 3 sedentary time (*β* = −.05, *p* = .10; 95% CI [−.11, .01]). As with MVPA and LPA, Time 1 self‐compassion did not exert a significant direct effect on Time 3 sedentary time (*β* = −.03, *p* = .41; 95% CI [−.11, .04]). These altogether led to a non‐significant indirect effect of Time 1 self‐compassion on Time 3 sedentary time via Time 2 barrier self‐efficacy (standardized estimate = −.00, *p* = .16; 95% CI [−.01, .00]).

## DISCUSSION

### Summary of findings

This study was designed to examine the longitudinal effect of state self‐compassion on physical activity behaviours (i.e., MVPA, LPA, sedentary time) and more importantly the mediation role of barrier self‐efficacy within the hypothesized effect. Compared to prior studies investigating the relationship between self‐compassion and physical activity levels (i.e., Hallion et al., 2019; Semenchuk et al., 2021; Zhang et al., 2023), the present research has several important advancements, including the adoption of a nine‐month three‐wave panel data design, the implementation of a state self‐compassion measure tailored for use in physical activity contexts and the recruitment of a well‐powered national representative sample. The results demonstrated consistent and positive correlations of baseline state self‐compassion on MVPA and LPA at Times 2 and 3. More importantly, the study data uncovered significant and positive indirect effects of baseline state self‐compassion on Time 3 MVPA and LPA via increased barrier self‐efficacy at Time 2, after controlling for autoregressive effects (e.g., Time 1 self‐compassion on Time 2 self‐compassion) and synchronous correlations of all study variables (e.g., covariance of self‐compassion and MVPA at Time 1, 2, or 3). The findings suggest state self‐compassion, especially one's self‐compassionate mind and approach to challenges and sufferings in physical activity, is facilitative to the engagement and maintenance of physical activity during the nine‐month study period, thanks to the superior barrier self‐efficacy as an outcome of high state self‐compassion.

### Research highlights

Besides the clear empirical support for the longitudinal effect of self‐compassion in promoting physical activity behaviours and the mediation role of barrier self‐efficacy within such an effect, there were several noteworthy points emerged from our data. First, despite the consistent prediction of self‐compassion on physical activity (e.g., correlation of baseline self‐compassion and Time 3 physical activity), the longitudinal effects of self‐compassion on physical activity behaviours (especially that of MVPA and LPA) were mediated by barrier self‐efficacy and the direct effect of baseline self‐compassion on physical activity behaviours at the nine‐month follow‐up was not significant after accounting for barrier self‐efficacy's mediation. This indicates that the role of self‐compassion as an antecedent of physical activity probably operates through increased efficacious belief and capacity of overcoming barriers and challenges (i.e., barrier self‐efficacy) in the context of physical activity. The findings extend the insights from Zhang et al.'s (2023) cross‐sectional study and suggest that self‐compassion training and practice maybe best tailored for addressing challenges or setbacks and overcoming barriers for promoting physical activity. Incorporating a general/trait not domain‐specific/state self‐compassion likely undermines its benefits in facilitating physical activity, which explains the lack of effect of self‐compassion on physical activity after controlling for physical activity‐related covariates (see Hallion et al., 2019; Semenchuk et al., 2021). Future intervention embedding self‐compassion for promoting physical activity should build an element of using a self‐compassionate mind^10^ as a coping or regulation strategy for getting through struggling, suffering, or difficult times (e.g., building self‐compassion to important pillars for developing resilience; see Kuchar et al., 2023) thus facilitating the adoption or maintenance desired physical activity.

Second, the results of the present research suggested that a self‐compassionate mind is more useful in facilitating high‐ not low‐intensity physical activity. Specifically, our data unveiled that the standardized indirect effect of state self‐compassion on MVPA was almost doubled compared to that on LPA (both were significant). It is possible that individuals tend to come across greater barriers or challenges in doing more vigorous types of physical activity (Biddle et al., 2011; Bodde & Seo, 2009; Rhodes et al., 2009; Sit et al., 2008) and our data revealed a greater frustration of time spent in MVPA compared to the relative stable time spent in LPA over the nine‐month study period. Self‐compassion is particularly useful when one is under a difficult time given its regulatory benefits in the context of physical activity (Cox et al., 2019; Huellemann et al., 2023; Semenchuk et al., 2018; Signore et al., 2021).

Last but not least, our data provided new evidence to support the cultivability of a self‐compassionate mind (Mosewich, 2020; Walton et al., 2022). Specifically, the magnitude of the autoregressive coefficients of Time 1 on Time 2 and Time 2 on Time 3 state self‐compassion appeared to be lower than that of barrier self‐efficacy over time, suggesting greater variance of state self‐compassion than barrier self‐efficacy during the nine‐month study period. Since self‐efficacy is a state, not a trait and is context‐specific and culturable (Bandura, 1977, 1997, 2004), the greater variance observed in self‐compassion infers that state self‐compassion, or a self‐compassionate mind in the context of physical activity, is at least as intervenable as or of similar variability as barrier self‐efficacy. Otherwise, one would have observed greater (not lower) autoregressive coefficients of state self‐compassion as an indication of stability over time. Future research would do well to explore the proximal influencers such as drivers contributing to or risk factors preventing the establishment of state self‐compassion in the context of physical activity, thus informing the design of self‐compassion practice for promoting regular physical activity.

### Limitations and other future directions

Despite a strong study design (e.g., three‐wave panel data collection, state self‐compassion measure, well‐powered representative sample), the present research is not without limitations. One limitation is the use of self‐report physical activity measures (i.e., IPAQ‐SF; Maddison et al., 2007) which may be prone to social desirability and recall error. However, the IPAQ‐based measure has been validated in multiple countries (see Craig et al., 2003; van Poppel et al., 2010) and importantly, we did not use the IPAQ‐SF to assess absolute levels of physical activity. Instead, we adopted the IPAQ‐SF as a relative measure of physical activity levels and controlled for both within‐ (e.g., change over time in physical activity) and between‐person (e.g., physical activity covariates) variations using the panel data design. Also, self‐report measures are probably the most feasible for assessing physical activity in a national representative sample. Nevertheless, in future studies of a smaller scale or using an intervention design, researchers would do well to adopt an accelerometer‐based measure of physical activity.^11^

In relation to the use of IPAQ‐SF, we were therefore restricted to assess physical activity levels but overlooked the types (e.g., the varying activities) and formats or settings (e.g., the delivery contexts) of physical activity. It is possible that self‐compassion may be more relevant or facilitative for certain types of physical activity or when the activities are organized in a specific format or setting. We encourage future research to collect wider physical activity data (e.g., types, contexts, etc.) and examine if a risk or facilitative factor may be altered.

Another potential limitation also relates to a measurement issue. Although we believe the adoption of a state self‐compassion measure was an advantage of the current study, the state self‐compassion measure we implemented could be further improved for future research and practice. More specifically, we did not change Neff et al.'s (2021) original state self‐compassion items but instructed participants to recall any physical activity‐related situation(s) they were experiencing at the time of data collection that might be painful, difficult, challenging, or made one feel inadequate in some way. Such an approach should allow participants to rate more precisely their state self‐compassion in the context of physical activity, but we acknowledge that the original state self‐compassion items may still be too general (not tailored for physical activity settings). The lack of specification of scale items for physical activity contexts may explain the relatively small effect of self‐compassion on physical activity behaviours. Future research should consider developing and validating a context‐specific measure of state self‐compassion for physical activity.

Moreover, our longitudinal data collection only observed dropout from Time 1 to 2 (i.e., 73% retention rate) but not from Time 2 to 3 (i.e., 100% retention rate). While the dropout rate from Time 1 to 2 was expected, the non‐dropout from Time 2 to 3 of the study may reveal potential confound or noises in data collection given the motivation of those retaining participants. That is, participants may be motivated to complete data collection so as to gain the incentive (i.e., £1 cash compensation per completed wave of data collection). However, we anticipated that if a certain portion of participants completed the data collection in a less careful or less serious way but just to earn the incentive, the effect observed in our analysis would have been underestimated due to increased error. As such, the significant findings we observed should be considered more conservative than expected,^12^ or the true effect size of a certain effect from our analysis may be larger than that achieved in our dataset. Regardless, we call for future researchers using an incentive to facilitate data collection to evaluate potential impacts such as incentives, as these could have a positive effect on the dataset and hypothesis testing.

Finally, although panel data design has been commonly used for causal interference in observational, non‐intervention studies (Finkel, 2020), randomized control trials (RCTs) or intervention/experimental designs with cautious control for confounders are considered more robust approaches for causal analysis (see Rohrer et al., 2022). This is because within‐person changes and between‐person differences are controlled via randomization, experimental manipulations, or intervention strategies in RCTs and intervention/experimental designs but only adjusted via statistical methods in panel data design. Therefore, future studies examining causality or a mediating effect would do well to consider RCTs or intervention/experimental designs not restricting to the panel data approach.

## CONCLUSION

Using three‐wave panel data from a well‐powered representative sample of the UK, we provided the first evidence of state self‐compassion's longitudinal effects on physical activity behaviours and more importantly the empirical support for barrier self‐efficacy being a mediating mechanism underlying such effects. After controlling for within‐ and between‐person variations, baseline state self‐compassion predicted physical activity levels at 5‐ and 9‐month follow‐up, of which barrier self‐efficacy at the 5‐month follow‐up mediated the effect of baseline state self‐compassion on physical activity at the 9‐month follow‐up. The findings, therefore, provide a strong support for the usefulness of a self‐compassionate mind in overcoming barriers for engaging in and maintaining physical activity. Future studies could consider accelerometer‐based physical activity measures and develop and validate more context‐specific state self‐compassion measures tailored for physical activity contexts. Researchers and practitioners should consider incorporating a self‐compassion component to interventions and education programmes for promoting physical activity.

## AUTHOR CONTRIBUTIONS

**Shuge Zhang:** Conceptualization; methodology; software; data curation; investigation; formal analysis; funding acquisition; visualization; project administration; writing – original draft; writing – review and editing. **Andy Pringle:** Conceptualization; methodology; investigation; resources; writing – review and editing; validation. **Clare Roscoe:** Conceptualization; methodology; investigation; validation; writing – review and editing; resources.

## FUNDING INFORMATION

This research received financial support from Research and Innovation, University of Derby, UK (awarded to the lead author; award code PSL2223‐0139).

## CONFLICT OF INTEREST STATEMENT

The authors do not have any conflict of interest.

## Data Availability

The data that support the findings of this study are available on request from the corresponding author. The data are not publicly available due to privacy or ethical restrictions.

## Footnotes

^1^
All dropouts occurred at the second wave of data collection (from Time 1 to Time 2), and no dropout was observed at the third/final wave of data collection (from Time 2 to Time 3).

^2^
After accounting for dropouts, the final sample (i.e., 654 completed data collection at Time 1, of which 475 also completed Time 2 and Time 3) remains sufficient to detect a relatively small, lagged effect (standardized coefficient = .15, power = .91), despite being marginal in statistical power for detecting an indirect effect of .02 (power = .72). In reality, the finally achieved power for detecting a .02 standardized indirect effect would be close to that obtained from the a priori power analysis (i.e., .92), because we used the *Full Information Maximum Likelihood* approach (Hirose et al., 2015) to address missing data (see Data Analysis section for details).

^3^
We used the term “self‐compassionate mind” adapting from Gilbert's compassionate mind but only focusing that an individual directs towards oneself (thus “self‐compassionate”) rather than that giving to or receiving from others (see also Zhang et al., 2024; Zhang & Boardley, 2022).

^4^
According to Neff et al.'s (2021, *p*.*124*), the assessment of state self‐compassion “… *was necessary that responses to items be focused on a single instance of suffering occurring in the moment (given that self‐compassion is a response to suffering)…*”. We made this adaption of instructions to better tackle state self‐compassion in the context of physical activity.

^5^
Despite using the representative sample screening function empowered by Prolific, we acknowledge that we were restricted to collect data in adult population and those who were not prevented from physical activity due to illness or any health concern(s). Nevertheless, we had built in the five ethnicity groups (i.e., Asian, Black, Mixed, White, Other; see https://www.ethnicity‐facts‐figures.service.gov.uk/uk‐population‐by‐ethnicity/national‐and‐regional‐populations/population‐of‐england‐and‐wales/latest/) adopted in the England and Wales 2021 Census to guide data collection, and our final sample by ethnicity was close to that of the England and Wales 2021 Census. We encourage future researchers using online or cloud‐sourcing data collection methods to select and collect appropriate data on demographics for monitoring and checking, or at least do not rely purely on the representative sample screening integrated in online and cloud‐sourcing platform.

^6^
Payment to participant is mandatory in Prolific, and we had constrained the payment to a minimum level based on average completion time (i.e., approximately 10 minutes) and the minimum hourly rate (i.e., £6). We excluded any participants who completed the survey too fast or stayed in the survey for too long time (i.e., 3SDs or more deviated away from the mean completion time). These excluded participants would not receive the incentive.

^7^
Thanks to the comment from an anonymised reviewer, we would like to take the opportunity to clarity for readers less familiar with *χ* ^2^ as a goodness‐of‐fit measure in case of any confusion. Specifically, a non‐significant *χ* ^2^ value is commonly considered as evidence of good‐fit model. However, a significant *χ* ^2^ is not necessarily evidence for poor model fit because the *χ* ^2^ is prone to false‐positive or Type 1 error as sample size increases. As such, guidance (e.g., Hu & Bentler, 1999) does not recommend relying on *χ* ^2^ to determine model fit but assess CFI, SRMR, and RMSEA instead. Nevertheless, it is still a common practice to report *χ* ^2^ as it can be particularly useful for comparing model fits such as via a Chi‐square difference test if needed. We direct interested readers to Kline (2016) for further reading.

^8^
The shared property of a lagged/direct effect (i.e., assessed by the regression coefficient) and an indirect effect (i.e., assessed by the multiplication of two regression coefficients for an indirect effect via one mediator) is noteworthy. However, we acknowledge that the completedly standardized estimate approach is not suitable when one or more binary/categorical variables are involved in a mediation model, and there are alternative effect size estimators for assessing mediation (see Preacher & Kelley, 2011).

^9^
For parsimony and focus on the main parameter estimations (i.e., lagged effect and indirect effect involved in the longitudinal mediation), we did not discuss details of standardized coefficient and its 95% CI of autoregressive effects and synchronous correlations in the main texts but display them in the figure of the tested models (i.e., Figures 1, 2, 3).

^10^
Thanks to the insight from an anonymised reviewer that regarding the conceptual position of self‐compassion (derived from Neff's perspective) and self‐compassionate mind (adapted from Gilbert's perspective). We would like to make a further note to our proposition of the two. In the present study and as a recommendation for future research, we argued that researchers and practitioners should acknowledge more fully not only the trait (relatively commonly known) but also the state (relatively overlooked) aspects of self‐compassion (see also Neff et al., 2021). In this context, the *self‐compassionate mind* is not only linked to one's trait self‐compassion but also can be fostered through proper training, education or intervention on the state aspects of self‐compassion, recognizing the social dynamic and the ‘skill’ nature or approach of the compassion towards oneself (see also Gilbert & Gordon, 2023). We argue that the use or incorporation of a self‐compassionate mind reflects more precisely Gilbert and Gordon's perspective of compassion as a skill and captures the state self‐compassion in Neff et al.'s conceptualisation, which can be a particularly useful coping or regulation strategy.

^11^
It is noteworthy that accelerometer‐based measures are not without limitations. We direct interested readers to Albrecht et al.'s (2022) study for an understanding of common issues and problems experienced by health researchers with experience of using accelerometers.

^12^
For further reassurance, we did a post‐hoc test to check for any potential disparity in results when using the completed sample (removing missing data at Times 2 and 3) compared to retaining the missing participants using the recommended full information maximum likelihood (FIML) approach. On this occasion, the statistical results generating from the two varied approaches were considerably identical (i.e., leading to the same conclusion of findings with similar magnitude of any observed effects). Nevertheless, we encourage future researchers to adopt the FIML approach or other validated missing data strategies when possible, as such practices generate greater statistical power thus the analysis is likely to produce more replicable and trustworthy results. However, specifical attention should be paid to situations when the analysis of complete dataset and the analysis of data with missing yielded meaningfully contradicting results.
